# *Garcinia kola* improves cognitive and motor function of a rat model of acute radiation syndrome in the elevated plus maze

**DOI:** 10.1093/braincomms/fcab170

**Published:** 2021-07-28

**Authors:** Nene Ahidjo, Leonard Ngarka, Paul F Seke Etet, Wepnyu Y Njamnshi, Leonard N Nfor, Michel K Mengnjo, Jonas G Basseguin Atchou, Edmond N Mouofo, Godwin Y Tatah, Faustin Dong A Zok, Bonaventure T Ngadjui, Wilfred Ngwa, Alfred K Njamnshi

**Affiliations:** 1 Neuroscience Laboratory, Faculty of Medicine and Biomedical Sciences, The University of Yaoundé I, Yaoundé, Cameroon; 2 Brain Research Africa Initiative (BRAIN), Geneva, Switzerland; 3 Brain Research Africa Initiative (BRAIN), Yaoundé, Cameroon; 4 Department of Neurology, Yaoundé Central Hospital, Yaoundé, Cameroon; 5 Center for Sustainable Health and Development, Garoua, Cameroon; 6 Department of Physiological Sciences and Biochemistry, University of Ngaoundéré, Garoua, Cameroon; 7 Department of Psychiatry, Yaoundé Military Hospital, Yaoundé, Cameroon; 8 Department of Neurology, CH Saint-Nazaire, Saint-Nazaire, France; 9 Radiotherapy Unit, Yaoundé General Hospital, Yaoundé, Cameroon; 10 Department of Pharmacology and Traditional Medicine, Faculty of Medicine and Biomedical Sciences, The University of Yaoundé I, Yaoundé, Cameroon; 11 Department of Radiation Oncology, Brigham and Women’s Hospital, Dana Farber Cancer Institute, Harvard Medical School, Boston, MA, USA

**Keywords:** *Garcinia kola*, Gamma radiation, anxiety-depression, acute radiation syndrome, motor and cognitive impairment

## Abstract

We reported recently that the elevated plus maze is a good tool for evaluating cognitive and motor functional changes in gamma-irradiated rats as a model for new drug evaluation and monitoring. The capacity of *Garcinia kola* to mitigate radiation-induced brain injury is currently unknown. We therefore assessed the effects of the neuroprotective medicinal plant *Garcinia kola*, on the cognitive and motor changes in this murine model of acute radiation syndrome. Wistar rats exposed once to an ionizing dose of Tc99m-generated Gamma radiation were treated with an ethyl acetate fraction of methanolic extract of *Garcinia kola* seeds (content of 100 mg/kg of extract) for 9 weeks. Cognitive and motor function indicators were assessed in the elevated plus maze in these animals and compared with irradiated control groups (vitamin C- and vehicle-treated groups) and the non-irradiated control rats. The irradiated control group displayed cachexia, shaggy and dirty fur, porphyrin deposits around eyes, decreased exploratory activity, reduced social interactions and a loss of thigmotaxis revealed by a marked decrease in rearing episodes and stretch attend posture episodes close to the walls of elevated plus maze closed arm, an increased central platform time, and decreases in open arm time and entries. This group further displayed a decrease in head dips and grooming episodes. Treatment with *Garcinia kola*, and in a lesser extent vitamin C, significantly prevented the body weight loss (*P* < 0.001) and mitigated the development of elevated plus maze signs of cognitive and motor affections observed in the irradiated control group (*P* < 0.05). Altogether, our data suggest for the first time that *Garcinia kola* seeds have protective properties against the development of cognitive and motor decline in the acute radiation syndrome-like context. Future studies are warranted to characterize the molecular mechanisms and neuronal networks of this action.

## Teaser

The effects of *G. kola* (a medicinal plant in African traditional medicine) seed extract on the cognitive and motor changes in the murine model of acute radiation syndrome were assessed. The extract was found to mitigate body weight loss, cognitive and motor impairment in irradiated rats better than vitamin C.

## Graphical Abstract

**Graphical Abstract:**
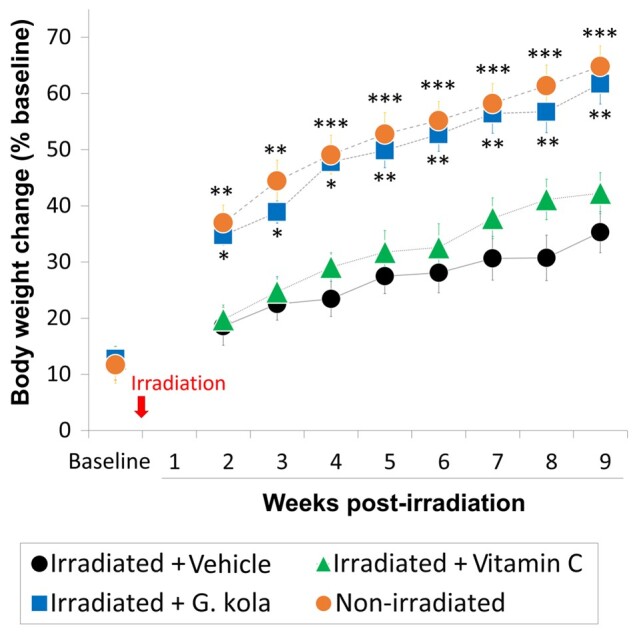

## Introduction

Human accidental exposure to large doses of ionizing radiations results in an intractable condition known as acute radiation syndrome, whose hallmark is characterized by a neurotoxicity syndrome marked by an inflammation-mediated encephalopathy and other debilitating and life-threatening pathologies resembling neurodegenerative disorders and ischaemic brain disease.^1^^,^^2^ The latter include pathologies such as those resulting from neuroinflammation and related neuronal loss, endothelial affections, and blood–brain barrier dysfunction.^3–8^ In large amounts, agents emitting Gamma radiations such as the commonly used imaging agent, technetium 99m (Tc99m) have been reported to cause brain damage in laboratory rodents,^9^^,^^10^ and acute radiation syndrome-like clinical signs were reported in such experimental exposure to Gamma radiations.^5–8^

Various studies using ethological tests, and particularly the elevated plus maze (EPM) paradigm,^11–13^ reported changes in rodent exploratory behaviour following experimental radiation-induced brain injury. The EPM paradigm is a commonly used ethological test based on conflict between rodent aversion for open brightly-lit spaces and tendency to explore a novel environment.^14–18^ We recently reported the changes in EPM indicators of CNS disease in rodents exposed to gamma radiation.^19^ We observed decreases in arm transitions, in the distance covered in the maze, in open arm time and entries, in head dipping and grooming episode number, in rearing episodes and stretch attend posture episodes, in the amount of urine released, and increases in faeces emitted by irradiated rats. These findings corroborate previous reports of increases in behavioural indicators of motor and cognitive affections in irradiated rodents.^11–13^ However, the capacity of *Garcinia kola* to mitigate radiation-induced brain injury is currently unknown.

Considering that therapeutic agents are urgently needed in the field for acute radiation syndrome^9^^,^^20^^,^^21^ but also for the neurological component of late complications of radiotherapy treatment,^2^^,^^22^^,^^23^ we assessed the effects of seeds of the medicinal plant *G.kola* (Guttiferae) on the development of cognitive and motor alterations typically observed in gamma-irradiated rats (GIR), a murine model of acute radiation syndrome. Generally referred to locally as ‘bitter kola’ because of its bitter taste or ‘male kola’ because of its other yet to be proven virtues, *G. kola* seeds have been used in African traditional medicine for treating various ailments, including liver and metabolic disorders, hepatitis, diarrhoea, laryngitis, bronchitis and gonorrhoea.^24^^,^^25^ The methanolic extract of *G. kola* seeds, in particular the ethyl acetate fraction, was reported to have neuroprotective and antioxidant properties.^25–29^

## Material and methods

### Animals and procedures

Forty-four male Wistar albino rats (194–200 g) were obtained from the animal house of the Faculty of Medicine and Biomedical Sciences (FMBS) of The University of Yaoundé I (Yaoundé, Cameroon) and housed at the Neuroscience Laboratory, FMBS, under natural day–night cycle, at 25°C. They had ad libitum access to normal rat chew and tap water.

Animals were randomly divided into four groups (*N* = 11 per group): a non-irradiated group given ethanol 50° (vehicle solution) (non-irradiated control group) and three groups irradiated once with gamma radiation.^19^ For 9 weeks after irradiation, the animals of the irradiated groups were given (daily, *per os*) *G. kola* extract in ethanol 50° (*G. kola*-treated group), ethanol 50° (irradiated control group) or vitamin C in distilled water (2.5 mg/kg)^30^ (vitamin C-treated or positive control group). Animals of the non-irradiated group were given the vehicle solution (non-irradiated control group). For each animal, the volume of solution to administer was calculated as follows:

$$\mathrm{Volume}\;(l)\;=\frac{\mathrm{Dose}\;(\mathrm{mg}/\mathrm{kg})\;\times \;\mathrm{Animal}\;\mathrm{weight}\;(\mathrm{kg})\;}{\mathrm{Ponderal}\;\mathrm{concentration}\;(\mathrm{mg}/\mathrm{ml})}$$

Cognitive and motor function impairment induced by irradiation and the effects of 9 weeks’ treatments were evaluated using the EPM behavioural test as earlier reported.^19^ Body weight was measured twice a week for each animal.

All the experimental procedures were approved by the institutional ethics committee. Animals were handled following ethical rules on the protection of animals used for scientific purposes, particularly European Commission directive (2010/63/EU).

### Plant processing and extract preparation

*Garciniakola* seeds were harvested during maturing period (August) in Bamenda, North West region of Cameroon. Seeds were authenticated by the National Herbarium of Cameroon and the department of Botany of the University of Yaoundé I, and a sample was stored (specimen N° 28837/HNC). Seed coats were peeled off and seeds were cut into small pieces and shade-dried at laboratory temperature. Dried seeds were ground into powder using a grinding mill. The powder (2500 g) was mixed and extracted with methanol at 65°C (5 h) using a Soxhlet extractor. Then, 100 ml of distilled water and 150 ml of ethyl acetate were added to the methanolic extract in a decantation ball. After 10 min, the dark organic phase was separated from the aqueous phase and collected. An additional 150 ml portion of ethyl acetate was added to the decantation ball and after 10 min, the organic phase was separated once again from the aqueous phase and collected. The process was repeated until the organic phase became less dark than the aqueous phase. The organic phases collected were mixed and dried using a rotatory evaporator at 70°C. The dried extract of *G. kola* (ethyl acetate fraction of methanolic extract, termed as *G. kola* extract in this manuscript) weighed 153.86 g (yield of the extraction: 6.2%). It was diluted in ethanol 50° and administered *per os* at 40 mg/ml to correspond to the content of a dose of 100 mg/kg of methanolic extract of *G. kola* seeds, considering that this dose of extract was reported to have strong antioxidant and neuroprotective effects in both our previous works^31^^,^^32^ and reports of other groups.^27^^,^^28^^,^^33^

### Gamma radiation exposure

The rats were irradiated by overexposure to large amount of technetium 99m (Tc99m) Gamma radiation as previously described.^19^ The targeted brain radiation absorption was 667 mGy, as absorptions ranging from 500 to 1000 mGy were reported to cause brain lesions.^9^^,^^10^ Briefly, 10 ml of a solution of pertechnetate was eluted from Tc99m-generator and a syringe of 1110 MBq of Gamma activity was prepared for each rat, corresponding to an absorbed dose of 667mGy (66.7Rad). The volume of radioactive Tc99m administered through the tail vein to each rat was 0.16 ml. Rat irradiation was done in the Radiotherapy unit of the Yaoundé General Hospital.

### EPM test

The EPM apparatus was elevated at 50 cm above the floor and consisted of two open arms (50 × 10 cm) crossed at a central platform (right angles) with two opposed arms of the same size enclosed by walls (40 cm high), with squares drawn on the floor. Each rat was placed on the central platform of the apparatus facing an open arm and was allowed to explore the maze for 5 min. Animal performance in the EPM was recorded using a computerized video recording system with a camera placed 150 cm above the centre of the apparatus. After 5 min, rats were returned to their home cage. After each trial, the walls and the floor of the arms and the floor of the central platform of the maze were cleaned with a 70% ethanol solution to prevent bias due to olfactory cues.

Video recordings were analysed offline using the Limelight Video Tracking System (Bilaney Consultants, Düsseldorf, Germany). Arm entries and the time in each arm were determined. An arm entry was counted when all the rat paws were in the arm. In addition, episodes of rearing (when an animal stood upright on hind limbs), head dipping (when an animal lowered its head over a side of the open arm towards the floor), grooming (when an animal licked and scratched itself for more than 3 s while stationary) and stretch attend posture (animal forward elongation of head and shoulders followed by retraction to original position) were counted. The total distance covered in the maze and the surface area of the urine released on the maze (puddles or streaks) floor were determined.

### Statistical analysis

Changes in body weight in the irradiated group given vehicle solution (irradiated control group) were compared to changes in the irradiated groups treated with *G. kola* extract or vitamin C, and to changes in the non-irradiated control group in each week post-treatment, using one-way ANOVA followed by Fisher's least significant difference (LSD) *post hoc* test. Differences in EPM performances at the end of the treatment were also compared between *G. kola*- and vitamin C-treated irradiated groups, the non-irradiated control group, and the irradiated control group using one-way ANOVA followed by LSD test. Differences with *P* < 0.05 were considered significant. Data were presented as mean ± standard deviation.

Our data are available as Supplementary material.

### Data availability statement

The data that support the findings of this study are available from the corresponding author, upon reasonable request.

## Results

### Animal condition

**Figure 1:**
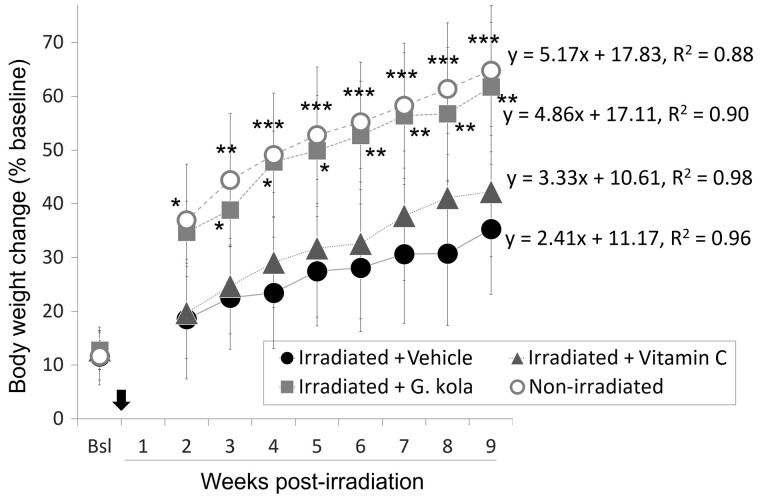
Effect of *G. kola* on irradiation-induced body weight changes. The black arrow indicates the irradiation day. Note the marked improvement in *G. kola*-treated group compared to the other irradiated groups. Bsl, baseline values. *N* = 11 per group. ANOVA + LSD test: *P < 0.05; **P < 0.01; ***P < 0.0001 versus irradiated group given the vehicle solution. Data mean ± standard deviation.

Treatment with *G. kola* improved the general animal condition and prevented the systemic disease signs observed in the irradiated control group, such as cachexia, porphyrin deposits around eyes, shaggy and dirty fur, decreased exploratory activity, and reduced social interactions. During handling every 3 days to change cage litter and to perform EPM tests, none of the animals expressed the common laboratory rodent marker of pain vocalization. Figure 1 shows body weight changes of irradiated animals receiving the vehicle solution, treatment with *G. kola*, treatment with vitamin C, and of non-irradiated animals. Irradiation slowed the increase in body weight (*y* = 2.41*x* + 11.17, *R*^2^ = 0.96) compared to the non-irradiated group (*y* = 5.17*x* + 17.83, *R*^2^ = 0.88), with marked differences from post-irradiation week 2 (*P* = 0.007). Improvements were observed in irradiated animals treated with *G. kola* (*y* = 4.86*x* + 17.11, *R*^2^ = 0.89), and in a lesser extent, vitamin C (*y* = 3.33*x* + 10.61, *R*^2^ = 0.98) (Fig. 1). The vitamin C effect was not statistically significant, unlike the *G. kola* effect, which was statistically significant from post-irradiation week 2 (*P* = 0.004) (Fig. 1). No statistically significant difference was observed between body weight changes of *G. kola*-treated and non-irradiated animals (Fig. 1).

### Arm entries and time in the EPM

**Figure 2:**
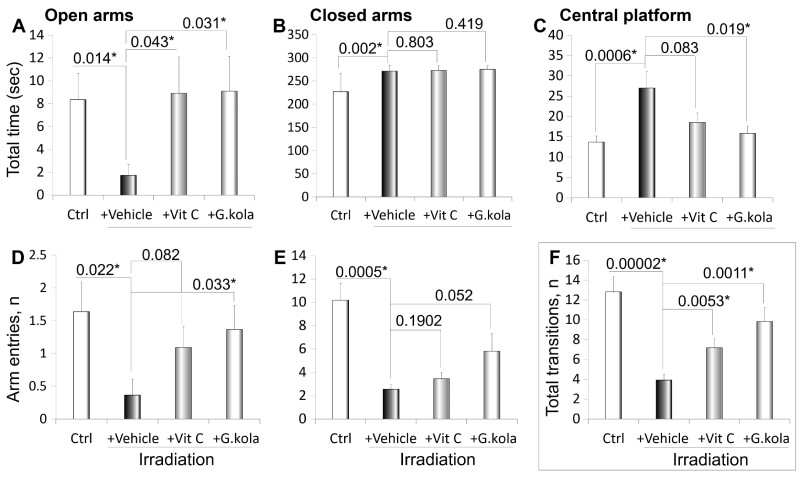
*Garcinia kola* effect on EPM arm entries and time. The parameters assessed in irradiated animals receiving the vehicle solution (ethanol 50°), treated with *G. kola*, treated with vitamin C (Vit C), and non-irradiated control animals (Ctrl) included: Open arm time (**A**), closed arm time (**B**), central platform time (**C**), open arm entries (**D**), closed arm entries (**E**) and total number of arm transitions (**F**). Numbers on top of bars are *P*-values of inter-group comparisons performed using ANOVA followed by LSD *post hoc* test. Note that most of the changes observed in vehicle group compared to non-irradiated animals (Ctrl) were mitigation in *G. kola*-treated, and in a lesser extent, vitamin C-treated group. *N* = 11 per group. *Statistically significant differences (*P* < 0.05, ANOVA + LSD test). Data mean ± standard deviation.

Figure 2 shows arm entries and time in the EPM of irradiated animals receiving the vehicle solution, treated with *G. kola*, treated with vitamin C, and of non-irradiated animals. Compared to the non-irradiated group, irradiated animals given the vehicle displayed significant decreases in open arm time (*P* = 0.014) (Fig. 2A), central platform time (*P* = 0.0006) (Fig. 2C), open arm entries (*P* = 0.022) (Fig. 2D), closed arm entries (*P* = 0.0005) (Fig. 2E) and total arm transitions (*P* = 0.0002) (Fig. 2F). Conversely, closed arm time was decreased (*P* = 0.002) (Fig. 2B). Vitamin C treatment prevented changes in open arm time (*P* = 0.043) and arm transitions (*P* = 0.0053) (Fig. 2A and F). On the other hand, *G. kola* treatment prevented or mitigated changes in open arm time (*P* = 0.031) (Fig. 2A), central platform time (*P* = 0.019) (Fig. 2C), arm entries (0.033) (Fig. 2D) and arm transitions (*P* = 0.0053) (Fig. 2F). No statistically significant difference was observed for these parameters between *G. kola*-treated and non-irradiated animals (Fig. 2A–F).

### Exploratory behaviour indicators in the EPM

**Figure 3:**
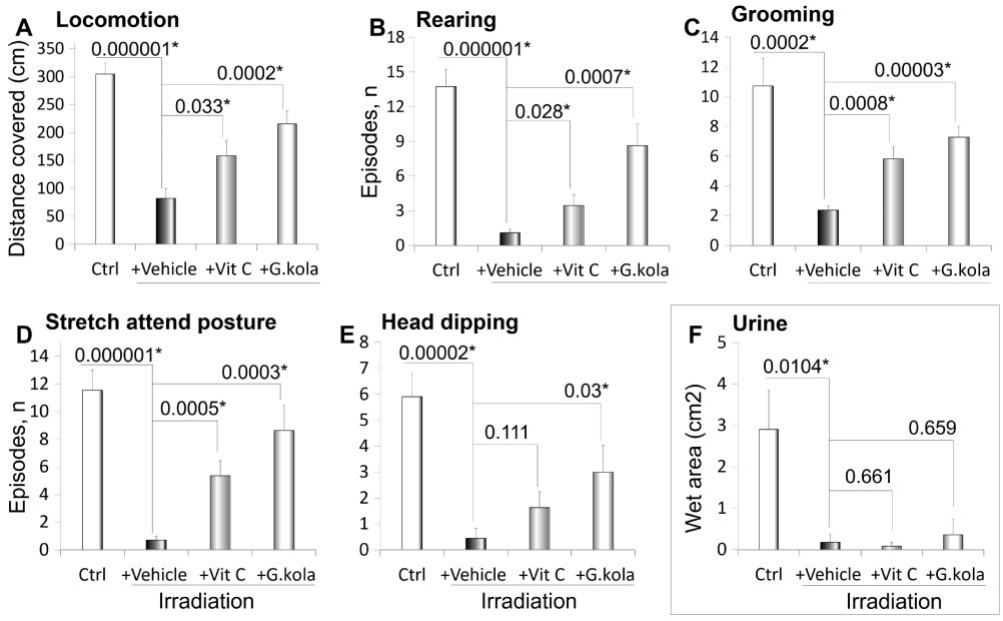
Exploratory behaviour indicators in the EPM. (**A**) Distance covered in the maze. (**B**) Number of rearing episodes. (**C**) Number of grooming episodes. (**D**) Number of stretch attend posture episodes. (**E**) Number of head dipping episodes. (**F**) Urine-wet area. Numbers on top of bars are *P*-values of inter-group comparisons performed using ANOVA + LSD test. Note the decreases of parameters in irradiated animals given the vehicle (ethanol 50°) compared to non-irradiated control animals (Ctrl), and the mitigation induced by *G. kola* treatment. Vit C, vitamin C-treated group. *N* = 11 per group. *Statistically significant differences (*P* < 0.05, ANOVA + LSD test). Data mean ± standard deviation.

Figure 3 shows the main exploratory behaviour indicators revealed by the EPM of irradiated animals receiving the vehicle solution, treated with *G. kola*, treated with vitamin C, and of non-irradiated animals. Compared to non-irradiated, irradiated animals given the vehicle displayed significant decreases in the distance covered in the maze (*P* = 0.000001) (Fig. 3A), the rearing episode number (*P* = 0.000001) (Fig. 3B), the grooming episode number (*P* = 0.0002) (Fig. 3C), the stretch attend posture episode number (*P* = 0.000001) (Fig. 3D), the head dipping episode number (*P* = 0.00002) (Fig. 3E) and the urine-wet area (*P* = 0.0104) (Fig. 3F). Vitamin C and *G. kola* treatments prevented decreases in the distance covered (*P* = 0.033 and *P* = 0.0002, respectively, compared to non-irradiated group) (Fig. 3A), the rearing episode number (*P* = 0.028 and *P* = 0.0007, respectively) (Fig. 3B), the grooming episode number (*P* = 0.0008 and *P* = 0.00003, respectively) (Fig. 3C), and the stretch attend posture episode number (*P* = 0.0005 and *P* = 0.0003, respectively) (Fig. 3D). *Garciniakola* treatment also prevented decreases in head dipping episode number (*P* = 0.03) (Fig. 3E).

## Discussion

The findings of this study suggest that *G. kola*, a medicinal plant with established neuroprotective and antioxidant properties,^27^^,^^28^^,^^31–33^ prevented the body weight loss and mitigated the typical cognitive and motor impairment in rats exposed once to a brain-damaging dose of Tc99m-generated Gamma radiation reported to cause. The results further highlighted the EPM as a good tool for evaluating cognitive and motor changes in the acute radiation syndrome-like context.

Notably, treatment with *G. kola* improved the animal general condition and prevented the systemic clinical signs previously reported in this model^12^^,^^13^ and also observed in the irradiated control group in the present study, such as cachexia, shaggy and dirty fur, porphyrin deposits around eyes, decreased exploratory activity, and reduced social interactions. Although, both *G. kola* and the antioxidant agent vitamin C increased the animal body weight, *G. kola* effect was stronger with non-irradiated group-like values, suggesting that *G. kola* effects were not only due to its well-established antioxidant properties.^25^^,^^27^^,^^32^^,^^33^

Furthermore, in this study, animals of the irradiated control group displayed marked alterations in the EPM indicators of motor and cognitive functions. Notably, a loss of thigmotaxis, a robust cognitive function indicator in rodents,^11–13^ was revealed in these animals by a marked decrease in rearing episodes and stretch attend posture episodes close to the walls of EPM closed arm. An increase in central platform time, which typically indicates an impairment in animal’s ability to choose the arm to explore,^14^^,^^15^ was observed in these animals. Moreover, decreases in arm transitions and in the distance covered in the maze, which are typical EPM indicators of cognitive and motor impairment,^14–18^ were also observed in animals of the irradiated control group in this study. On the other hand, *G. kola* treatment, and in a lesser extent, vitamin C treatment, prevented the increase in central platform time and mitigated the decreases in arm transitions, in the distance covered in the maze, and in rearing episodes and stretch attend posture episodes close to the walls of EPM closed arm. These findings suggest that *G. kola* treatment prevented or mitigated the development of cognitive and motor impairment in GIR.

Besides, decreases were observed in open arm time and entries, in head dipping episode number, and in the amount of urine released during the test, i.e. in EPM standard indicators of anxiety-like mood,^14–18^ in irradiated control group. Irradiated control group animals also had shaggy and dirty fur, and displayed a marked decrease in the number of grooming episodes, all indicators of depression-like mood.^5^^,^^7^ Interestingly, treatment of irradiated animals with either vitamin C or *G. kola* significantly mitigated the development of these pathologic signs. With an overall stronger response than vitamin C, treatment of irradiated animals with *G. kola* prevented decreases in open arm time and in the number of grooming episodes, mitigated the decreases in arm entries and head dipping episode number, and increased slightly the amount of urine released during the test. Altogether, our observations in GIR corroborate previous reports from behavioural studies in irradiated rodents where decreases in motor activity and increases in indicators of anxiety-like mood were observed.^11–13^ Finally, our data suggest that *G. kola* treatment improved the general condition of the animals as well as motor and cognitive functions in GIR.

Further studies are needed to elucidate the mechanisms by which the observed action by *G. kola* is exerted. Such studies could evaluate the effects at varying doses of G. *kola* extracts. Future studies will also extend the current assessment to evaluate the potential of G. *kola* in mitigating toxicities due to external beam radiotherapy of brain tumours or metastasis, which could be highly valuable in radiation oncology.

## Conclusion

We assessed the effect of the ethyl acetate extract of seeds of *G. kola* on EPM cognitive and motor indicators in rats exposed once to a brain-damaging dose of Tc99m-generated Gamma radiation, a murine model of acute radiation syndrome. The *G. kola* seed extract prevented the body weight loss and mitigated the typical EPM indicators of cognitive and motor impairment in GIR better than vitamin C, suggesting that these effects were only partly due to the established antioxidant properties of seeds of this medicinal plant. Further fractionation and mechanism studies are warranted to unravel the active principles and pathways accounting for the mitigation of decreases in cognitive and motor functions by *G. kola* seed extract in acute radiation syndrome-like context.

## Supplementary material

Supplementary material is available at *Brain Communications* online.

## Supplementary Material

fcab170_Supplementary_Data

## Abbreviations

EPM =: elevated plus maze
GIR =: Gamma-irradiated rats
LSD test =: least significant difference test
