# Supplementary material for: Garcinia kola improves cognitive and motor function of a rat model of acute radiation syndrome in the elevated plus maze
Source: Brain Commun. 2021 Jul 28;3(3):fcab170. doi: 10.1093/braincomms/fcab170 (PMC8361417; doi:10.1093/braincomms/fcab170)
Supplement: fcab170_Supplementary_Data [file fcab170_supplementary_data.zip › Original Submission BRAINCOM-2021-155.pdf]

OXFORD UNIVERSITY PRESS | Brain Communications

Garcinia kola improves cognitive and motor function of a rat model of acute radiation syndrome in the elevated plus maze

Journal:	*Brain Communications*
Manuscript ID	BRAINCOM-2021-155
Manuscript Type:	Original Article
Date Submitted by the Author:	03-May-2021

Complete List of Authors:	Ahidjo, Nene; The University of Yaounde I, Neuroscience Laboratory, Faculty of Medicine and Biomedical Sciences; Brain Research Africa Initiative (BRAIN), Translational Neuroscience<br>Ngarka, Leonard; The University of Yaounde I, Neuroscience Laboratory, Faculty of Medicine and Biomedical Sciences; Brain Research Africa Initiative (BRAIN)<br>Seke Etet, Paul; Center for Sustainable Health and Development, Department of Health; University of Ngaoundéré, 5 Department of Physiological Sciences and Biochemistry,<br>Njamnshi, Wepnyu; The University of Yaounde I, Neuroscience Laboratory, Faculty of Medicine and Biomedical Sciences; Brain Research Africa Initiative, Translational Neuroscience<br>Nfor, Leonard; The University of Yaounde I, Neuroscience Laboratory, Faculty of Medicine and Biomedical Sciences; Brain Research Africa Initiative, Neuroscience; Central Hospital of Yaounde, Neurology<br>Mengnjo, Michel; University of Yaounde I, Neuroscience Laboratory, Faculty of Medicine and Biomedical Sciences; Brain Research Africa Initiative, Neuroscience; Central Hospital of Yaounde, Neurology<br>Atchou, Jonas Guy; University of Yaounde I, Neuroscience Laboratory, Faculty of Medicine and Biomedical Sciences; Brain Research Africa Initiative, Neuroscience; Yaoundé Military Hospital, Psychiatry<br>Mouofo, Edmond; University of Yaounde I, Neuroscience Laboratory, Faculty of Medicine and Biomedical Sciences<br>Tatah, Godwin; University of Yaounde I, Neuroscience Laboratory, Faculty of Medicine and Biomedical Sciences; Brain Research Africa Initiative, Neuroscience; CH Saint Nazaire, Neurology<br>Dong A Zok, Faustin; Yaoundé General Hospital, Radiotherapy<br>Ngadjui, Bonaventure ; University of Yaounde I, Department of Pharmacology and Traditional Medicine, Faculty of Medicine and Biomedical Sciences<br>Ngwa, Wilfred; Dana Farber Cancer Institute, Harvard Medical School, Radiation Oncology<br>Njamnshi, Alfred; Brain Research Africa Initiative (BRAIN), Neuroscience Research; University of Yaounde I, Neuroscience Lab, Faculty of Medicine and Biomedical Sciences

Keywords:	Garcinia kola;, Gamma radiation;, anxiety-depression;, acute radiation syndrome;, elevated plus maze;, motor and cognitive impairment.

SCHOLARONE™
Manuscripts

Garcinia kola improves cognitive and motor function of a rat model of acute radiation syndrome in the elevated plus maze

Nene AHIDJO[1,2], Leonard NGARKA[1,2,3], Paul F. SEKE ETET[4,5], Wepnyu Y. NJAMNSHI[1,2], Leonard N. NFOR[1,2,3], Michel K. MENGNJO[1,2,3], Jonas G. BASSEGUIN ATCHOU[1,2,6], Edmond N. MOUOFO[1], Godwin Y. TATAH[1,2,7], Faustin DONG A ZOK[8], Bonaventure T. NGADJUI[9], Wilfred NGWA[10], Alfred K. NJAMNSHI[1,2,3]*.

[1] Neuroscience Laboratory, Faculty of Medicine and Biomedical Sciences, The University of Yaoundé I, Yaoundé, Cameroon.

[2] Brain Research Africa Initiative (BRAIN), Geneva, Switzerland & Yaoundé, Cameroon,

[3] Department of Neurology, Yaoundé Central Hospital, Yaoundé, Cameroon.

[4] Center for Sustainable Health and Development, Garoua, Cameroon.

[5] Department of Physiological Sciences and Biochemistry, University of Ngaoundéré, Garoua, Cameroon.

[6] Department of Psychiatry, Yaoundé Military Hospital, Yaoundé, Cameroon.

[7] Department of Neurology, CH Saint-Nazaire, Saint-Nazaire, France.

[8] Radiotherapy unit, Yaoundé General Hospital, Yaoundé, Cameroon.

[9] Department of Pharmacology and Traditional Medicine, Faculty of Medicine and Biomedical Sciences, The University of Yaoundé I, Yaoundé, Cameroon.

[10] Department of Radiation Oncology, Brigham and Women's Hospital, Dana Farber Cancer Institute, Harvard Medical School, Boston, USA.

***Corresponding author:**

Prof. Alfred K. NJAMNSHI[1]

Neuroscience Laboratory, Faculty of Medicine, University of Yaoundé I, & Brain Research Africa Initiative (BRAIN), PO Box 25625, Yaoundé, Cameroon.

Email alfred.njamnshi@brainafrica.org

For Review Only

ORCID 000-0003-0052-1511

For Review Only

Abstract

Background: We reported recently that the elevated plus maze (EPM) is a good tool for evaluating cognitive and motor functional changes in gamma-irradiated rats as a model for new drug evaluation and monitoring. The capacity of *Garcinia kola* to mitigate radiation-induced brain injury is currently unknown.

Rationale: We therefore assessed the effects of the neuroprotective medicinal plant *Garcinia kola*, on the cognitive and motor changes in this murine model of acute radiation syndrome.

Methods: Wistar rats exposed once to an ionizing dose of Tc99m-generated Gamma radiation were treated with an ethyl acetate fraction of methanolic extract of *G. kola* seeds (content of 100 mg/kg of extract) for 9 weeks. Cognitive and motor function indicators were assessed in the elevated plus maze (EPM) in these animals and compared with irradiated control groups (vitamin C- and vehicle-treated groups) and the non-irradiated control rats.

Results: The irradiated control group displayed cachexia, shaggy and dirty fur, porphyrin deposits around eyes, decreased exploratory activity, reduced social interactions and a loss of thigmotaxis revealed by a marked decrease in rearing episodes and stretch attend posture episodes close to the walls of EPM closed arm, an increased central platform time, and decreases in open arm time and entries. This group further displayed a decrease in head dips and grooming episodes. Treatment with *G. kola*, and in a lesser extent vitamin C, significantly prevented the body weight loss ($P < 0.001$) and mitigated the development of EPM signs of cognitive and motor affections observed in the irradiated control group ($P < 0.05$).

Conclusion: Altogether, our data suggest for the first time that *G. kola* seeds have protective properties against the development of cognitive and motor decline in the acute radiation

For Review Only

syndrome-like context. Future studies are warranted to characterize the molecular mechanisms and neuronal networks of this action.

Keywords: *Garcinia kola;* Gamma radiation; anxiety-depression; acute radiation syndrome; elevated plus maze; motor and cognitive impairment.

For Review Only

1. Introduction

Human accidental exposure to large doses of ionizing radiations result in an intractable condition known as acute radiation syndrome, whose hallmark is characterised by a neurotoxicity syndrome marked by an inflammation-mediated encephalopathy and other debilitating and life-threatening pathologies resembling neurodegenerative disorders and ischemic brain disease [1, 2]. The latter include pathologies such as those resulting from neuroinflammation and related neuronal loss, endothelial affections, and blood-brain barrier dysfunction [3-8]. In large amounts, agents emitting Gamma radiations such as the commonly used imaging agent, technetium 99m (Tc99m) have been reported to cause brain damage in laboratory rodents [9, 10], and acute radiation syndrome-like clinical signs were reported in such experimental exposure to Gamma radiations [5-8].

Various studies using ethological tests, and particularly the elevated plus maze (EPM) paradigm [11-13], reported changes in rodent exploratory behaviour following experimental radiation-induced brain injury. The EPM paradigm is a commonly used ethological test based on conflict between rodent aversion for open brightly-lit spaces and tendency to explore a novel environment [14-18]. We recently reported the changes in EPM indicators of central nervous system disease in rodents exposed to gamma radiation [19]. We observed decreases in arm transitions, in the distance covered in the maze, in open arm time and entries, in head dipping and grooming episode number, in rearing episodes and stretch attend posture episodes, in the amount of urine released, and increases in faeces emitted by irradiated rats. These findings corroborate previous reports of increases in behavioural indicators of motor and cognitive affections in irradiated rodents [11-13]. However, the capacity of *Garcinia kola* to mitigate radiation-induced brain injury is currently unknown.

For Review Only

Considering that therapeutic agents are urgently needed in the field for acute radiation syndrome [9, 20, 21] but also for the neurological component of late complications of radiotherapy treatment [2, 22, 23], we assessed the effects of seeds of the medicinal plant *Garcinia kola* (Guttiferae) on the development of cognitive and motor alterations typically observed in gamma-irradiated rats, a murine model of acute radiation syndrome. Generally referred to locally as “bitter kola” because of its bitter taste or “male kola” because of its other yet to be proven virtues, *G. kola* seeds have been used in African traditional medicine for treating various ailments, including liver and metabolic disorders, hepatitis, diarrhea, laryngitis, bronchitis and gonorrhea [24, 25]. The methanolic extract of *G. kola* seeds, in particular the ethyl acetate fraction, was reported to have neuroprotective and antioxidant properties [25-29].

2. Material and Methods

2.1. Animals and procedures

Forty-four male Wistar albino rats (194-200g) were obtained from the animal house of the Faculty of Medicine and Biomedical Sciences (FMBS) of the University of Yaoundé I (Yaoundé, Cameroon) and housed at the Laboratory of Neuroscience, FMBS, under natural day-night cycle, at 25°C. They had ad libitum access to normal rat chew and tap water.

Animals were randomly divided into four groups (N = 11 per group): three groups irradiated once with gamma radiation [19] and a non-irradiated group. For 9 weeks after irradiation, the animals of the irradiated groups were given (daily, *per os*) *G. kola* extract in ethanol 50° (*G. kola*-treated group), ethanol 50° (irradiated control group), or vitamin C in distilled water (2.5 mg/kg) [30] (vitamin C-treated or positive control group). Animals of the

For Review Only

non-irradiated group were given the vehicle solution (non-irradiated control group). For each animal, the volume of solution to administer was calculated as follows:

$$\text{Volume (l)} = \frac{\text{Dose (mg/kg) x Animal Weight (kg)}}{\text{Ponderal Concentration (mg/ml)}}$$

Cognitive and motor function impairment induced by irradiation and the effects of 9 weeks' treatments were evaluated using the elevated plus maze (EPM) behavioral test as earlier reported [19]. Body weight was measured twice a week for each animal.

2.2. Ethical Statement

The research protocol was reviewed and approved by the Institutional Review Board (IRB) of the Faculty of Medicine and Biomedical Sciences of The University of Yaoundé I. All the experimental procedures were carried out as approved by the IRB committee. Animals were handled following ethical rules on the protection of animals used for scientific purposes, particularly European Commission directive (2010/63/EU).

For Review Only

2.3. Plant processing and extract preparation

G. Kola seeds were harvested during maturing period (August) in Bamenda, North West region of Cameroon. Seeds were authenticated by the National Herbarium of Cameroon and the department of Botany of the University of Yaoundé I, and a sample was stored (specimen N° 28837/HNC). Seed coats were peeled off and seeds were cut into small pieces and shade-dried at laboratory temperature. Dried seeds were ground into powder using a grinding mill. The powder (2,500 g) was mixed and extracted with methanol at 65°C (5 hours) using a Soxhlet extractor. Then, 100 ml of distilled water and 150 ml of ethyl acetate were added to the methanolic extract in a decantation ball. After 10 min, the dark organic phase was separated from the aqueous phase and collected. An additional 150 ml portion of

ethyl acetate was added to the decantation ball and after 10 min, the organic phase was separated once again from the aqueous phase and collected. The process was repeated until the organic phase became less dark than the aqueous phase. The organic phases collected were mixed and dried using a rotatory evaporator at 70°C. The dried extract of *G. kola* (ethyl acetate fraction of methanolic extract, termed as *G. kola* extract in the remainder of this manuscript) weighed 153.86 g (yield of the extraction: 6.2%). It was diluted in ethanol 50° and administered *per os* at 40 mg/ml to correspond to the content of a dose of 100 mg/kg of methanolic extract of *G. kola* seeds, considering that this dose of extract was reported to have strong antioxidant and neuroprotective effects in both our previous works [31, 32] and reports of other groups [27, 28, 33].

For Review Only

2.4. Gamma radiation exposure

The rats were irradiated by overexposure to large amount of technetium 99m (Tc99m) Gamma radiation as previously described [19]. The targeted brain radiation absorption was 667mGy, as absorptions ranging from 500 to 1000 mGy were reported to cause brain lesions [9, 10]. Briefly, 10ml of a solution of pertechnetate was eluted from Tc99m-generator and a syringe of 1110MBq of Gamma activity was prepared for each rat, corresponding to an absorbed dose of 667mGy (66.7Rad). The volume of radioactive Tc99m administered through the tail vein to each rat was 0.16mL. Rat irradiation was done in the Radiotherapy unit of the Yaoundé General Hospital.

2.5. Elevated Plus Maze test

The EPM apparatus was elevated at 50 cm above the floor and consisted of two open arms (50 x 10 cm) crossed at a central platform (right angles) with two opposed arms of the same size enclosed by walls (40 cm high), with squares drawn on the floor. Each rat was placed on

the central platform of the apparatus facing an open arm and was allowed to explore the maze for 5 minutes. Animal performance in the EPM was recorded using a computerized video recording system with a camera placed 150 cm above the center of the apparatus. After 5 minutes, rats were returned to their home cage. After each trial, the walls and the floor of the arms and the floor of the central platform of the maze were cleaned with a 70% ethanol solution to prevent bias due to olfactory cues.

Video recordings were analysed offline using the Limelight Video Tracking System (Bilaney Consultants, Düsseldorf, Germany). Arm entries and the time in each arm were determined. An arm entry was counted when all the rat paws were in the arm. In addition, episodes of rearing (when an animal stood upright on hind limbs), head dipping (when an animal lowered its head over a side of the open arm toward the floor), grooming (when an animal licked and scratched itself for more than 3 sec while stationary), and stretch attend posture (animal forward elongation of head and shoulders followed by retraction to original position) were counted. The total distance covered in the maze and the surface area of the urine released on the maze (puddles or streaks) floor were determined.

2.6. Data analysis

Differences in body weight (all along the treatment period) and EPM performances (at the end of the treatment) between the irradiated groups administered with *G. kola*, with vitamin C, or with ethanol 50°, and the non-irradiated group were compared using ANOVA followed by LSD post hoc test. Differences with $p < 0.05$ were considered significant. Data were presented as means ± SEM.

For Review Only

3. Results

3.1. Animal clinical condition

Treatment with *G. kola* improved the general animal clinical condition and prevented the systemic disease signs observed in the irradiated control group, such as cachexia, porphyrin deposits around eyes, shaggy and dirty fur, decreased exploratory activity, and reduced social interactions. Figure 1 shows body weight changes of irradiated animals receiving the vehicle solution, treatment with *G. kola*, treatment with vitamin C, and of non-irradiated animals. Irradiation slowed the increase in body weight ($y = 2.41x + 11.17$, $R^2 = 0.96$) compared to the non-irradiated group ($y = 5.17x + 17.83$, $R^2 = 0.88$), with marked differences from post-irradiation week 2 ($p = 0.007$). Improvements were observed in irradiated animals treated with *G. kola* ($y = 4.86x + 17.11$, $R^2 = 0.89$), and in a lesser extent, vitamin C ($y = 3.33x + 10.61$, $R^2 = 0.98$) (Figure 1). The vitamin C effect was not statistically significant, unlike the *G. kola* effect, which was statistically significant from post-irradiation week 2 ($p = 0.004$) (Figure 1). No statistically significant difference was observed between body weight changes of *G. kola*-treated and non-irradiated animals (Figure 1).

3.3. Arm entries and time in the EPM

Figure 2 shows arm entries and time in the elevated plus maze of irradiated animals receiving the vehicle solution, treated with *G. kola*, treated with vitamin C, and of non-irradiated animals. Compared to the non-irradiated group, irradiated animals given the vehicle displayed significant decreases in open arm time ($p = 0.014$) (Figure 2A), central platform time ($p = 0.0006$) (Figure 2C), open arm entries ($p = 0.022$) (Figure 2D), closed arm entries ($p = 0.0005$) (Figure 2E), and total arm transitions ($p = 0.0002$) (Figure 2F). Conversely, close arm time was decreased ($p = 0.002$) (Figure 2B). Vitamin C treatment

For Review Only

prevented changes in open arm time ($p = 0.043$) and arm transitions ($p = 0.0053$) (Figure 2A,F). On the other hand, *G. kola* treatment prevented or mitigated changes in open arm time ($p = 0.031$) (Figure 2A), central platform time ($p = 0.019$) (Figure 2C), arm entries (0.033) (Figure 2D), and arm transitions ($p = 0.0053$) (Figure 2F). No statistically significant difference was observed for these parameters between *G. kola*-treated and non-irradiated animals (Figure 2A-F).

3.4. Exploratory behaviour indicators in the EPM

Figure 3 shows the main exploratory behavior indicators revealed by the EPM of irradiated animals receiving the vehicle solution, treated with *G. kola*, treated with vitamin C, and of non-irradiated animals. Compared to non-irradiated, irradiated animals given the vehicle displayed significant decreases in the distance covered in the maze ($p = 0.000001$) (Figure 3A), the rearing episode number ($p = 0.000001$) (Figure 3B), the grooming episode number ($p = 0.0002$) (Figure 3C), the stretch attend posture episode number ($p = 0.000001$) (Figure 3D), the head dipping episode number ($p = 0.00002$) (Figure 3E), and the urine-wet area ($p = 0.0104$) (Figure 3F). Vitamin C and *G. kola* treatments prevented decreases in the distance covered ($p = 0.033$ and $p = 0.0002$, respectively, compared to non-irradiated group) (Figure 3A), the rearing episode number ($p = 0.028$ and $p = 0.0007$, respectively) (Figure 3B), the grooming episode number ($p = 0.0008$ and $p = 0.00003$, respectively) (Figure 3C), and the stretch attend posture episode number ($p = 0.0005$ and $p = 0.0003$, respectively) (Figure 3D). *G. kola* treatment also prevented decreases in head dipping episode number ($p = 0.03$) (Figure 3E).

For Review Only

4. Discussion

The findings of the present study suggest that *G. kola,* a medicinal plant with established neuroprotective and antioxidant properties [27, 28, 31-33], prevented the body weight loss and mitigated the typical cognitive and motor impairment in rats exposed once to a brain-damaging dose of Tc99m-generated Gamma radiation reported to cause. The results further highlighted the EPM as a good tool for evaluating cognitive and motor changes in the acute radiation syndrome-like context.

Notably, treatment with *G. kola* improved the animal general condition and prevented the systemic clinical signs previously reported in this model [12, 13] and also observed in the irradiated control group in the present study, such as cachexia, shaggy and dirty fur, porphyrin deposits around eyes, decreased exploratory activity, and reduced social interactions. Although, both *G. kola* and the antioxidant agent vitamin C increased the animal body weight, *G. kola* effect was stronger with non-irradiated group-like values, suggesting that *G. kola* effects were not only due to its well-established antioxidant properties [25, 27, 32, 33].

Furthermore, in this study, animals of the irradiated control group displayed marked alterations in the EPM indicators of motor and cognitive functions. Notably, a loss of thigmotaxis, a robust cognitive function indicator in rodents [11-13], was revealed in these animals by a marked decrease in rearing episodes and stretch attend posture episodes close to the walls of EPM closed arm. An increase in central platform time, which typically indicates an impairment in animal's ability to choose the arm to explore [14, 15], was observed in these animals. Moreover, decreases in arm transitions and in the distance covered in the maze, which are typical EPM indicators of cognitive and motor impairment [14-18], were also observed in animals of the irradiated control group in this study. On the

For Review Only

other hand, *G. kola* treatment, and in a lesser extent, vitamin C treatment, prevented the increase in central platform time and mitigated the decreases in arm transitions, in the distance covered in the maze, and in rearing episodes and stretch attend posture episodes close to the walls of EPM closed arm. These findings suggest that *G. kola* treatment prevented or mitigated the development of cognitive and motor impairment in gamma-irradiated rats.

Besides, decreases were observed in open arm time and entries, in head dipping episode number, and in the amount of urine released during the test, i.e. in EPM standard indicators of anxiety-like mood [14-18], in irradiated control group. Irradiated control group animals also had shaggy and dirty fur, and displayed a marked decrease in the number of grooming episodes, all indicators of depression-like mood [5, 7]. Interestingly, treatment of irradiated animals with either vitamin C or *G. kola* significantly mitigated the development of these pathologic signs. With an overall stronger response than vitamin C, treatment of irradiated animals with *G. kola* prevented decreases in open arm time and in the number of grooming episodes, mitigated the decreases in arm entries and head dipping episode number, and increased slightly the amount of urine released during the test. Altogether, our observations in gamma-irradiated rats corroborate previous reports from behavioural studies in irradiated rodents where decreases in motor activity and increases in indicators of anxiety-like mood were observed [11-13]. Finally, our data suggest that *G. kola* treatment improved the general condition of the animals as well as motor and cognitive functions in gamma-irradiated rats.

Our study had some limitations. We recognise that our experimental model involving total body acute radiation exposure with Tc99m [19], is not a good model for brain injury from intensity-modulated radiation therapy (IMRT) for primary brain tumours nor whole

For Review Only

brain radiation in some cases for brain metastases in human clinical practice. Nevertheless, as a model for the acute radiation syndrome, our approach appears to be satisfactory especially considering that all the experimental animal groups in this study underwent the same method of irradiation, thus permitting valid comparisons. Lastly, the challenges of performing experiments with animals in our context did not allow us to repeat the experiments.

Further studies are needed to elucidate the mechanisms by which the observed action by *G. kola* is exerted. Such studies could evaluate the effects at varying doses of G. *Kola* extracts. Future studies will also extend the current assessment to evaluate the potential of G. *kola* in mitigating toxicities due to external beam radiotherapy of brain tumours or metastasis, which could be highly valuable in radiation oncology.

For Review Only

Conclusion

We assessed the effect of the ethyl acetate extract of seeds of *G. kola* on EPM cognitive and motor indicators in rats exposed once to a brain-damaging dose of Tc99m-generated Gamma radiation, a murine model of acute radiation syndrome. The *G. kola* seed extract prevented the body weight loss and mitigated the typical EPM indicators of cognitive and motor impairment in gamma-irradiated rats better than vitamin C, suggesting that these effects were only partly due to the established antioxidant properties of seeds of this medicinal plant. Further fractionation and mechanism studies are warranted to unravel the active principles and pathways accounting for the mitigation of decreases in cognitive and motor functions by *G. kola* seed extract in the acute radiation syndrome-like context.

Acknowledgements

The experimental and recording equipment were acquired and made available to the Neuroscience Laboratory by Brain Research Africa Initiative (BRAIN) – www.brainafrica.org Specials thanks are due to the Nuclear Medicine Service of Yaoundé General Hospital (Cameroon). This work is part of the PhD thesis project of the lead author.

Conflict of interest disclosure

Authors declare no competing financial interest.

For Review Only

References

1. Happold, C., et al., *Anticoagulation for radiation-induced neurotoxicity revisited.* J Neurooncol, 2008. 90(3): p. 357-62.
2. Behin, A. and J.Y. Delattre, *Complications of radiation therapy on the brain and spinal cord.* Semin Neurol, 2004. 24(4): p. 405-17.
3. Erkkinen, M.G., M.O. Kim, and M.D. Geschwind, *Clinical Neurology and Epidemiology of the Major Neurodegenerative Diseases.* Cold Spring Harb Perspect Biol, 2018. 10(4).
4. Kalaria, R.N., R. Akinyemi, and M. Ihara, *Stroke injury, cognitive impairment and vascular dementia.* Biochim Biophys Acta, 2016. 1862(5): p. 915-25.
5. Song, X., et al., *Silibinin ameliorates anxiety/depression-like behaviors in amyloid beta-treated rats by upregulating BDNF/TrkB pathway and attenuating autophagy in hippocampus.* Physiol Behav, 2017. 179: p. 487-493.
6. Pinz, M.P., et al., *Current advances of pharmacological properties of 7-chloro-4-(phenylselanyl) quinoline: Prevention of cognitive deficit and anxiety in Alzheimer's disease model.* Biomed Pharmacother, 2018. 105: p. 1006-1014.
7. Khan, A., et al., *Matrine ameliorates anxiety and depression-like behaviour by targeting hyperammonemia-induced neuroinflammation and oxidative stress in CCl4 model of liver injury.* Neurotoxicology, 2019. 72: p. 38-50.
8. Guignet, M., et al., *Persistent behavior deficits, neuroinflammation, and oxidative stress in a rat model of acute organophosphate intoxication.* Neurobiol Dis, 2020. 133: p. 104431.
9. Duran, A., E. Duran, and J. Castro, *[Diagnosis of brain death scintigraphy with HMPAO-TC99m].* Neurologia, 2003. 18(7): p. 389.
10. Zolotov, V.A., et al., *[Experimental study of the mechanism of local radiation damage to the brain by means of penetration of 99mTc through the hematoencephalic barrier].* Med Radiol (Mosk), 1982(7): p. 53-8.
11. Norton, S., P. Mullenix, and B. Culver, *Comparison of the structure of hyperactive behavior in rats after brain damage from x-irradiation, carbon monoxide and pallidal lesions.* Brain Res, 1976. 116(1): p. 49-67.
12. Peng, Y., et al., *Blockade of Kv1.3 channels ameliorates radiation-induced brain injury.* Neuro Oncol, 2014. 16(4): p. 528-39.
13. Thabet, N.M. and E.M. Moustafa, *Protective effect of rutin against brain injury induced by acrylamide or gamma radiation: role of PI3K/AKT/GSK-3beta/NRF-2 signalling pathway.* Arch Physiol Biochem, 2018. 124(2): p. 185-193.
14. Vuralli, D., et al., *Behavioral and cognitive animal models in headache research.* J Headache Pain, 2019. 20(1): p. 11.
15. Castanheira, L., et al., *Anxiety Assessment in Pre-clinical Tests and in Clinical Trials: A Critical Review.* Curr Top Med Chem, 2018. 18(19): p. 1656-1676.
16. Seke Etet, P.F., et al., *Evaluation of the safety of conventional lighting replacement by artificial daylight.* J Microsc Ultrastruct, 2017. 5(4): p. 206-215.
17. Padurariu, M., et al., *Describing some behavioural animal models of anxiety and their mechanistics with special reference to oxidative stress and oxytocin relevance.* Int J Vet Sci Med, 2017. 5(2): p. 98-104.
18. Djiogue, S., et al., *Memory and exploratory behavior impairment in ovariectomized Wistar rats.* Behav Brain Funct, 2018. 14(1): p. 14.

For Review Only

19. Njamnshi, A.K., et al., *Characterization of the Cognitive and Motor Changes Revealed by the Elevated Plus Maze in an Experimental Rat Model of Radiation‐Induced Brain Injury.* Adv. Biomed. Res., 2020.
20. Wilkinson, G.S., et al., *Mortality among plutonium and other radiation workers at a plutonium weapons facility.* Am J Epidemiol, 1987. 125(2): p. 231-50.
21. Beral, V., et al., *Mortality of employees of the Atomic Weapons Establishment, 1951-82.* Bmj, 1988. 297(6651): p. 757-70.
22. Pasquier, D., et al., *Hyperbaric oxygen therapy in the treatment of radio-induced lesions in normal tissues: a literature review.* Radiother Oncol, 2004. 72(1): p. 1-13.
23. Kelsey, C.R., et al., *Assessing neurotoxicity from the low-dose radiation component of radiosurgery using magnetic resonance spectroscopy.* Neuro Oncol, 2010. 12(2): p. 145-52.
24. Icheku, V., F. Onianwah, and A. Nwulia, *A descriptive cross-sectional study on various uses and outcomes of Garcinia kola among people of Oshimili North in the Delta State of Nigeria.* Ayu, 2018. 39(3): p. 132-138.
25. Djague, F., et al., *Garcinia kola (Heckel) and Alchornea cordifolia (Schumach. & Thonn.) Müll. Arg. from Cameroon possess potential antisalmonellal and antioxidant properties.* PLoS One, 2020. 15(8): p. e0237076.
26. Farombi, E.O., et al., *Garcinia kola seed biflavonoid fraction (Kolaviron), increases longevity and attenuates rotenone-induced toxicity in Drosophila melanogaster.* Pestic Biochem Physiol, 2018. 145: p. 39-45.
27. Adedara, I.A., et al., *Garcinia kola seed ameliorates renal, hepatic, and testicular oxidative damage in streptozotocin-induced diabetic rats.* Pharm Biol, 2015. 53(5): p. 695-704.
28. Farombi, E.O., et al., *Hepatic, testicular and spermatozoa antioxidant status in rats chronically treated with Garcinia kolaseed.* J Ethnopharmacol, 2013. 146(2): p. 536-42.
29. Okoko, T., *In vitro antioxidant and free radical scavenging activities of Garcinia kola seeds.* Food Chem Toxicol, 2009. 47(10): p. 2620-3.
30. Kim, J.H., et al., *The Therapeutic Effect of Vitamin C in an Animal Model of Complex Regional Pain Syndrome Produced by Prolonged Hindpaw Ischemia-Reperfusion in Rats.* Int J Med Sci, 2017. 14(1): p. 97-101.
31. Seke Etet, P.F., et al., *Garcinia kola seeds may prevent cognitive and motor dysfunctions in a type 1 diabetes mellitus rat model partly by mitigating neuroinflammation.* J Complement Integr Med, 2017. 14(3).
32. Farahna, M., et al., *Garcinia kola aqueous suspension prevents cerebellar neurodegeneration in long-term diabetic rat - a type 1 diabetes mellitus model.* J Ethnopharmacol, 2017. 195: p. 159-165.
33. Abarikwu, S.O., *Kolaviron, a natural flavonoid from the seeds of Garcinia kola, reduces LPS-induced inflammation in macrophages by combined inhibition of IL-6 secretion, and inflammatory transcription factors, ERK1/2, NF-κB, p38, Akt, p-c-JUN and JNK.* Biochim Biophys Acta, 2014. 1840(7): p. 2373-81.

For Review Only

Figures

Figure 1. Effect of *G. kola* on irradiation-induced body weight changes.

The black arrow indicates the irradiation day. Note the marked improvement in *G. kola* – treated group compared to the other irradiated groups. **Bsl:** baseline values. N=11 per group. ANOVA+LSD test: *p<0.05 **p<0.01 *** p<0.0001

Figure 2. *G. kola* effect on EPM arm entries and time.

The parameters assessed in irradiated animals receiving the vehicle solution (**dH2O**), treated with *G. kola*, treated with vitamin C (**Vit C**), and non-irradiated animals (**Ctrl**) included: Open arm time (**A**), closed arm time (**B**), central platform time (**C**), open arm entries (**D**), closed arm entries (**E**), and total number of arm transitions (**F**). Numbers on top of bars are p-values of inter-group comparisons performed using ANOVA followed by LSD post hoc test. Note that most of the changes observed in vehicle group compared to non-irradiated animals (**Ctrl**) were mitigation in G. kola-treated, and in a lesser extent, vitamin C-treated group. N=11 per group. *Statistically significant differences.

For Review Only

Figure 3. Exploratory behavior indicators in the EPM.

A. Distance covered in the maze. **B.** Number of rearing episodes. **C**. Number of grooming episodes. **D**. Number of stretch attend posture episodes. **E**. Number of head dipping episodes. **F**. Urine-wet area. Numbers on top of bars are p-values of inter-group comparisons performed using ANOVA +LSD test. Note the decreases of parameters in irradiated animals given the vehicle (dH2O) compared to non-irradiated animals (Ctrl), and the mitigation induced by *G. kola* treatment. **Vit C:** vitamin C-treated group. N=11 per group. *Statistically significant differences.

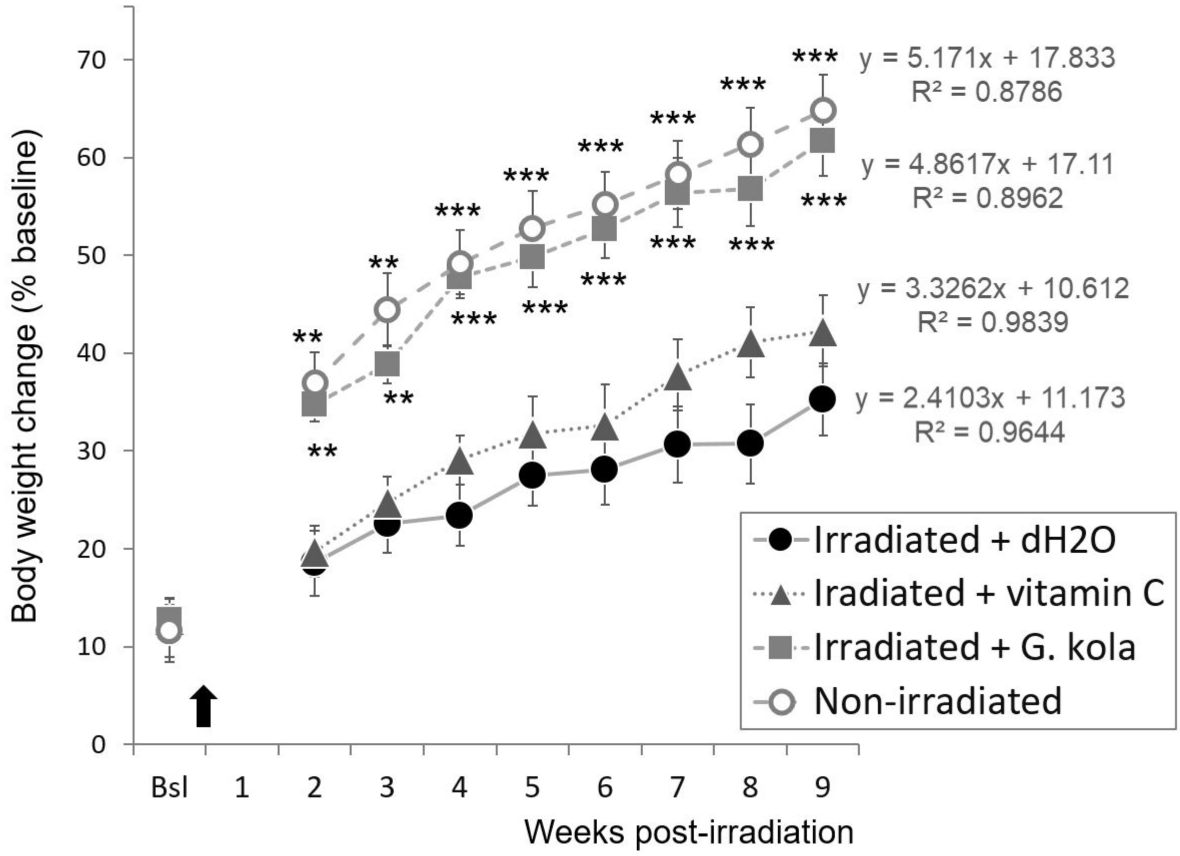

Fig 1

view Only

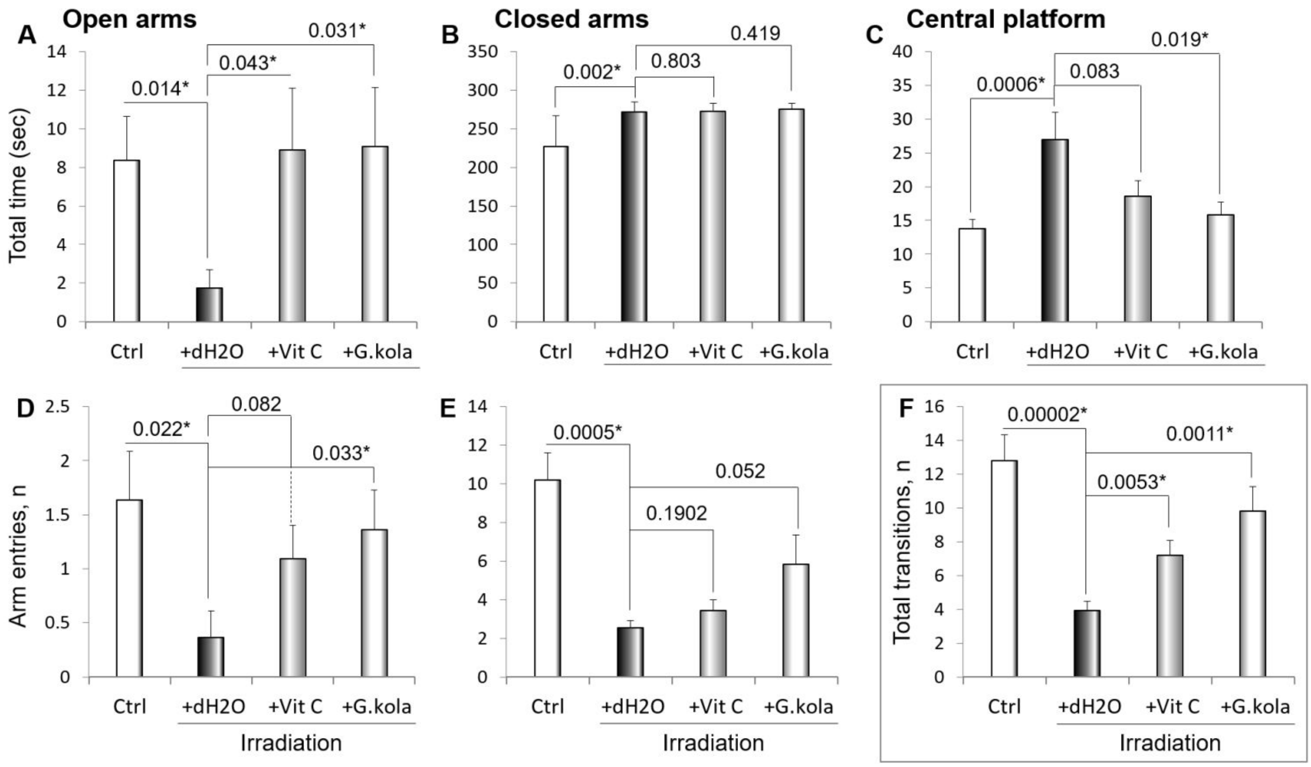

Fig 2

For Peer Review Only

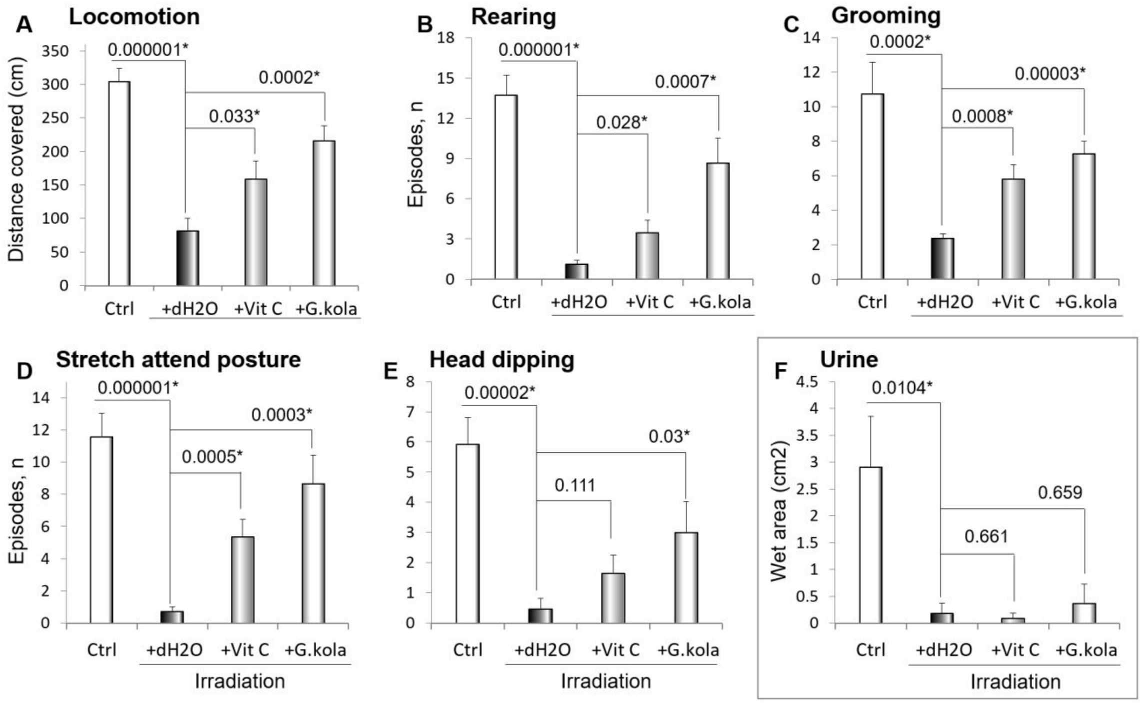

Fig 3

Review Only

Abbreviated Summary (Translational Significance):

We suggested the elevated plus maze (EPM) as a good tool for evaluating cognitive and motor changes in gamma-irradiated rats (GRR) as a model for new drug evaluation and monitoring. *Garcinia kola* is a common medicinal plant in African traditional medicine but its capacity to mitigate radiation-induced brain injury is unknown. We therefore assessed the effects of *G. kola* seeds, on the cognitive and motor changes in the murine model of acute radiation syndrome. The *G. kola* extract mitigated body weight loss and typical EPM cognitive and motor impairment in GGR better than vitamin C, indicating that these effects were only partly due to the established antioxidant properties of *G. kola*. Future studies have to characterize the molecular mechanisms and neuronal networks of this action and determine the potential of *G. kola* in mitigating toxicities due to external beam radiotherapy of brain tumours/metastasis, with a high impact in radiation neuro-oncology.

For Review Only

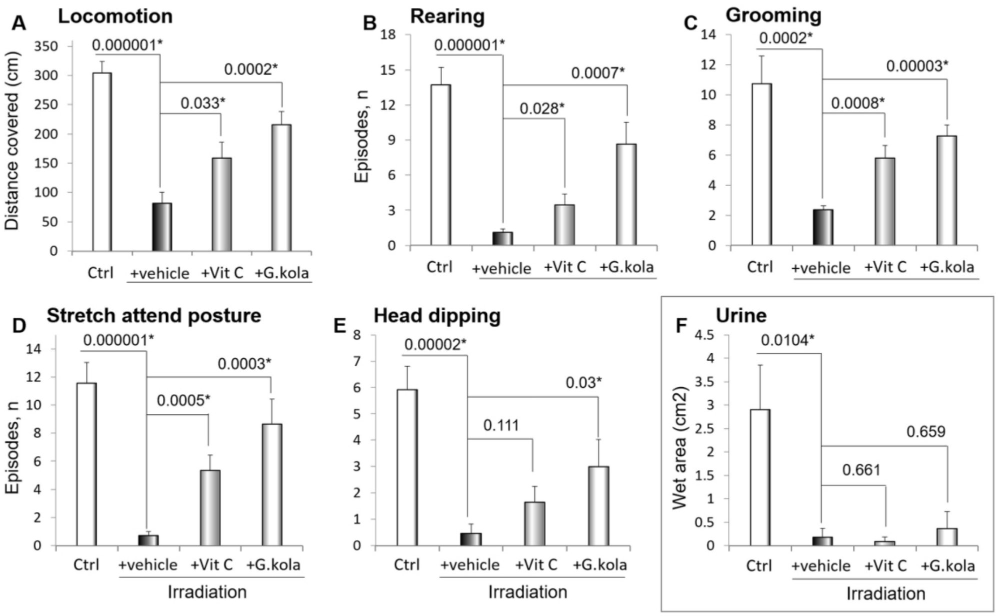

Graphical Abstract

161x99mm (300 x 300 DPI)

ARRIVE

The ARRIVE Guidelines Checklist

Animal Research: Reporting In Vivo Experiments

Carol Kilkenny[1], William J Browne[2], Innes C Cuthill[3], Michael Emerson[4] and Douglas G Altman[5]

[1]The National Centre for the Replacement, Refinement and Reduction of Animals in Research, London, UK, [2]School of Veterinary Science, University of Bristol, Bristol, UK, [3]School of Biological Sciences, University of Bristol, Bristol, UK, [4]National Heart and Lung Institute, Imperial College London, UK, [5]Centre for Statistics in Medicine, University of Oxford, Oxford, UK.

	ITEM	RECOMMENDATION	Section/ Paragraph
Title	1	Provide as accurate and concise a description of the content of the article as possible.	Done
Abstract	2	Provide an accurate summary of the background, research objectives, including details of the species or strain of animal used, key methods, principal findings and conclusions of the study.	Done
INTRODUCTION			
Background	3	a. Include sufficient scientific background (including relevant references to previous work) to understand the motivation and context for the study, and explain the experimental approach and rationale.<br>b. Explain how and why the animal species and model being used can address the scientific objectives and, where appropriate, the study's relevance to human biology.	Done
Objectives	4	Clearly describe the primary and any secondary objectives of the study, or specific hypotheses being tested.	Done
METHODS			
Ethical statement	5	Indicate the nature of the ethical review permissions, relevant licences (e.g. Animal [Scientific Procedures] Act 1986), and national or institutional guidelines for the care and use of animals, that cover the research.	Done
Study design	6	For each experiment, give brief details of the study design including:<br>a. The number of experimental and control groups.<br>b. Any steps taken to minimise the effects of subjective bias when allocating animals to treatment (e.g. randomisation procedure) and when assessing results (e.g. if done, describe who was blinded and when).<br>c. The experimental unit (e.g. a single animal, group or cage of animals).<br>A time-line diagram or flow chart can be useful to illustrate how complex study designs were carried out.	Done
Experimental procedures	7	For each experiment and each experimental group, including controls, provide precise details of all procedures carried out. For example:<br>a. How (e.g. drug formulation and dose, site and route of administration, anaesthesia and analgesia used [including monitoring], surgical procedure, method of euthanasia). Provide details of any specialist equipment used, including supplier(s).<br>b. When (e.g. time of day).<br>c. Where (e.g. home cage, laboratory, water maze).<br>d. Why (e.g. rationale for choice of specific anaesthetic, route of administration, drug dose used).	Done
Experimental animals	8	a. Provide details of the animals used, including species, strain, sex, developmental stage (e.g. mean or median age plus age range) and weight (e.g. mean or median weight plus weight range).<br>b. Provide further relevant information such as the source of animals, international strain nomenclature, genetic modification status (e.g. knock-out or transgenic), genotype, health/immune status, drug or test naïve, previous procedures, etc.	Done

The ARRIVE guidelines. Originally published in *PLoS Biology*, June 2010[1]

Housing and husbandry	9	Provide details of:<br>a. Housing (type of facility e.g. specific pathogen free [SPF]; type of cage or housing; bedding material; number of cage companions; tank shape and material etc. for fish).<br>b. Husbandry conditions (e.g. breeding programme, light/dark cycle, temperature, quality of water etc for fish, type of food, access to food and water, environmental enrichment).<br>c. Welfare-related assessments and interventions that were carried out prior to, during, or after the experiment.	Done
Sample size	10	a. Specify the total number of animals used in each experiment, and the number of animals in each experimental group.<br>b. Explain how the number of animals was arrived at. Provide details of any sample size calculation used.<br>c. Indicate the number of independent replications of each experiment, if relevant.	Done<br><br>Not applicable
Allocating animals to experimental groups	11	a. Give full details of how animals were allocated to experimental groups, including randomisation or matching if done.<br>b. Describe the order in which the animals in the different experimental groups were treated and assessed.	Done
Experimental outcomes	12	Clearly define the primary and secondary experimental outcomes assessed (e.g. cell death, molecular markers, behavioural changes).	Done
Statistical methods	13	a. Provide details of the statistical methods used for each analysis.<br>b. Specify the unit of analysis for each dataset (e.g. single animal, group of animals, single neuron).<br>c. Describe any methods used to assess whether the data met the assumptions of the statistical approach.	Done
RESULTS			
Baseline data	14	For each experimental group, report relevant characteristics and health status of animals (e.g. weight, microbiological status, and drug or test naïve) prior to treatment or testing. (This information can often be tabulated).	Done
Numbers analysed	15	a. Report the number of animals in each group included in each analysis. Report absolute numbers (e.g. 10/20, not 50%[2]).<br>b. If any animals or data were not included in the analysis, explain why.	Done
Outcomes and estimation	16	Report the results for each analysis carried out, with a measure of precision (e.g. standard error or confidence interval).	Done
Adverse events	17	a. Give details of all important adverse events in each experimental group.<br>b. Describe any modifications to the experimental protocols made to reduce adverse events.	Done
DISCUSSION			
Interpretation/ scientific implications	18	a. Interpret the results, taking into account the study objectives and hypotheses, current theory and other relevant studies in the literature.<br>b. Comment on the study limitations including any potential sources of bias, any limitations of the animal model, and the imprecision associated with the results[2].<br>c. Describe any implications of your experimental methods or findings for the replacement, refinement or reduction (the 3Rs) of the use of animals in research.	Done
Generalisability/ translation	19	Comment on whether, and how, the findings of this study are likely to translate to other species or systems, including any relevance to human biology.	Done
Funding	20	List all funding sources (including grant number) and the role of the funder(s) in the study.	Done

References:
1. Kilkenny C, Browne WJ, Cuthill IC, Emerson M, Altman DG (2010) Improving Bioscience Research Reporting: The ARRIVE Guidelines for Reporting Animal Research. *PLoS Biol* 8(6): e1000412. doi:10.1371/journal.pbio.1000412
2. Schulz KF, Altman DG, Moher D, the CONSORT Group (2010) CONSORT 2010 Statement: updated guidelines for reporting parallel group randomised trials. *BMJ* 340:c332.

For Review Only
