# Supplementary material for: Garcinia kola improves cognitive and motor function of a rat model of acute radiation syndrome in the elevated plus maze
Source: Brain Commun. 2021 Jul 28;3(3):fcab170. doi: 10.1093/braincomms/fcab170 (PMC8361417; doi:10.1093/braincomms/fcab170)
Supplement: fcab170_Supplementary_Data [file Original Submission Decision Letter.pdf › Original Submission Decision Letter.pdf]

Dear Prof. Njamnshi,

Thank you for your recent submission BRAINCOM-2021-155 entitled "Garcinia kola improves cognitive and motor function of a rat model of acute radiation syndrome in the elevated plus maze" to Brain Communications.

Your manuscript has been reviewed, and while the reviewers and editorial team appreciate many aspects of the paper, they raise some concerns that you will need to address in order for the paper to be considered for publication. Both reviewers raise concerns about interpreting cognitive data in light of systemic effects of irradiation (feeding and pain).

If you can thoroughly address these concerns, I invite you to submit a revised manuscript and a response to each of the reviewers' comments. The reviewers’ comments are included at the bottom of this message.

In addition to the reviewers' comments, please note our editorial comments added below to comply with our requirements for presentation of scientific data at Brain Communications.

Please format your revised manuscript according to our Instructions to Authors https://academic.oup.com/braincomms/pages/General_Instructions – a summary of key points is attached to help with this.

To revise your manuscript, click the link below or log into https://mc.manuscriptcentral.com/braincom and enter your Author Centre, where you will find your manuscript title listed under "Manuscripts with Decisions." Under "Actions," click on "Create a Revision." Your manuscript number will be appended to denote a revision.

*** PLEASE NOTE: This is a two-step process. After clicking on the link, you will be directed to a webpage to confirm. ***

https://mc.manuscriptcentral.com/braincom?URL_MASK=45bd34f774234d72b92c9eaa6b1cc8e2

Please upload a revised manuscript with changes tracked with track changes or in coloured text.

When submitting your revised manuscript, you will be asked to respond to the reviewer(s) comments. Please be as specific as possible in your response to all of the comments.

The editorial team at Brain Communications aims to increase transparency in scientific publishing and to demonstrate the value that peer review brings to the scientific process. Please therefore consider whether you are willing to have reviewer(s)’ comments on your paper and your response published as supplementary data with the paper if it is accepted. This is entirely optional and will only be done if both the authors and reviewers agree. While submitting your revision there will be an opportunity to review or update the answer you gave previously, if necessary.

IMPORTANT: Your original files are available to you when you upload your revised manuscript. Please delete any redundant files before completing the submission.

Because we are trying to facilitate timely publication of manuscripts submitted to Brain Communications, we would appreciate your revised manuscript as soon as possible. If it is not possible for you to resubmit within 3 months, the option to submit a revision will expire, and we would then consider your revised paper as a new submission. However, if you need a little more time, please get in touch and we can extend this deadline for you.

Once again, thank you for submitting your manuscript to Brain Communications and I look forward to receiving your revision.

Best wishes

Professor Tara Spires-Jones
Founding Editor, Brain Communications
Tara.Spires-Jones@ed.ac.uk

Reviewer(s)' Comments to Author:

Reviewer 1:

Comments to the Author
The study by Ahidjo et al presents some intriguing findings related to the potential antioxidant and neuroprotective properties of Garcinia kola extracts. However, the manuscript has some important inconsistencies between methods and results' section, presents inadequate and even opaque statistical analysis, and the authors put forward exacerbated claims related to the key finding.
Inconsistencies
In the method section the 4 experimental groups are detailed as:
(1) G. kola-treated group (Irradiation and G. kola extract in ethanol 50°)
(2) Irradiated control group ((Irradiation and ethanol 50°)
(3) Vitamin C treated group (Irradiation and vitamin C in water)
(4) Non-irradiated control group (treated with unknown identity vehicle)
However, in the results and the figure legends the 'Irradiated control group' is stated to have received dH2O (not ethanol). This has major implications on the interpretation of data presented.
If there is no group with irradiation and ethanol the entirety of the effects seen and attributed to Garcinia kola extracts could be simply due to alcohol, and it's well known pain relief properties.
Moreover, the nature of the vehicle that the control (non-irradiated) group received is not clear. Nothing is mentioned in the results or figure legends to clarify this.
Lastly, the classification of vitamin C group as positive control is not evident. Positive control for what exactly?
Statistical analysis reporting
The statistical methods used are not clear. A simple mention of 'ANOVA' and 'LSD post-hoc' does not say much about the analysis applied. Also reporting just p values is not informative. Given that the results have been plotted as mean with bars indicating SEM (as stated in methods), some p values are clearly confusing. For example in Figure 2B the comparison between control and dH2O group has statistical significance (P=0.002) when the error bars from control group clearly reach the mean value of dH2O group. This is impossible.
Exacerbated claims
Data presented here suggest that the clinical image of the animals which receive irradiation is very poor. They barely move, or groom their fur, and they reduce the amount they eat. Especially the last two phenotypes are well known sign of distress and pain for laboratory animal (and more specifically rodents). Given this, I would urge caution when making any claims about cognitive effects of this irradiation procedure. Virtually every behavioural task used in rodents, and specifically EPM, relies on ambulatory activity or the subjects. Therefore, the most parsimonious explanation of behavioural phenotypes after radiation poisoning is distress/pain that leads to reduced movement.

Reviewer 2:

Reviewer name: O Ishola

Comments to the Author
The manuscript titled Garcinia kola improves cognitive and motor function of a rat model of acute radiation syndrome in the elevated plus maze is well written and conceived but I have some on the use of EPM for cognitive decline, although, EPM could be used to measure cognitive functions but the parameters observed for in this study are basically anxiety-like and motor functions [Time spent in central platform could not be use as yardstick for memory function but that of decision making]. the figures are too tiny thus, increase their sizes. it is necessary for the authors to assay for biochemical markers to ascertain the level of neurodegeneration having exposed the whole body to gamma irradiation, any impact on feeding. it will be advisable to expose only the head to radiation while shielding the rest of the body

Editorial Office comments:

-The graphical abstract supplied has a lot of graphs and text that may not render well in our table of contents, could you please provide a simpler graphical abstract? A graphical abstract is a visual representation of the central finding or methodology of the paper. It will be displayed in the article and in the table of contents, and used to promote your article on Twitter. Authors should strive to make the graphical abstract informative, interesting, visually appealing, and straightforward (no caption needed, no abbreviations except gene/protein names). Please upload it as a separate high resolution image file (tiff preferred). The technical requirements for graphical abstracts are:
-size of the submitted image: 1200 pixels square at 300dpi
-font of the text: Arial 12-16 points (smaller fonts will not be legible online)

-Please include the page numbers corresponding to the items listed in the checklist.

-Please include a paragraph about data availability in the 'Materials and Methods' section. We strongly encourage data sharing either as supplementary information or uploads to repositories which you can link in the paper.
