# Supplementary material for: Garcinia kola improves cognitive and motor function of a rat model of acute radiation syndrome in the elevated plus maze
Source: Brain Commun. 2021 Jul 28;3(3):fcab170. doi: 10.1093/braincomms/fcab170 (PMC8361417; doi:10.1093/braincomms/fcab170)
Supplement: fcab170_Supplementary_Data [file Original Submission Response To Reviewers.pdf › Original Submission Response To Reviewers.pdf]

Response to Reviewers' Comments

BRAINCOM-2021-155: "Garcinia kola improves cognitive and motor function of a rat model of acute radiation syndrome in the elevated plus maze"

Reviewer: 1

Comments to the Author

The study by Ahidjo et al presents some intriguing findings related to the potential antioxidant and neuroprotective properties of *Garcinia kola* extracts. However, the manuscript has some important inconsistencies between methods and results' section, presents inadequate and even opaque statistical analysis, and the authors put forward exacerbated claims related to the key finding.

Inconsistencies

1. In the method section the 4 experimental groups are detailed as:
   (1) G. kola-treated group (Irradiation and G. kola extract in ethanol 50°)
   (2) Irradiated control group ((Irradiation and ethanol 50°)
   (3) Vitamin C treated group (Irradiation and vitamin C in water)
   (4) Non-irradiated control group (treated with unknown identity vehicle)

   However, in the results and the figure legends the 'Irradiated control group' is stated to have received dH2O (not ethanol). This has major implications on the interpretation of data presented.

   If there is no group with irradiation and ethanol the entirety of the effects seen and attributed to *Garcinia kola* extracts could be simply due to alcohol, and its well-known pain relief properties.

The typo was corrected. The manuscript and the figures were edited to state clearly that the vehicle solution was ethanol 50°. Distilled water was used only to dissolve vitamin C.

2. Moreover, the nature of the vehicle that the control (non-irradiated) group received is not clear. Nothing is mentioned in the results or figure legends to clarify this.

The manuscript now clearly states that the vehicle solution was ethanol 50°, used also for the non-irradiated group.

3. Lastly, the classification of vitamin C group as positive control is not evident. Positive control for what exactly?

Vitamin C was the positive control for neuroprotection, as the study was assessing whether *G. kola* extract would protect the rats from irradiation-induced neurodegeneration. Vitamin C is a well-established neuroprotective agent in various animal models of neurodegenerative conditions and diseases, partly thanks to its potent antioxidant effects. Some relatively recent interesting studies supporting this include:

https://pubmed.ncbi.nlm.nih.gov/26831257/
https://pubmed.ncbi.nlm.nih.gov/25701025/
https://pubmed.ncbi.nlm.nih.gov/29423921/

Statistical analysis reporting

4. The statistical methods used are not clear. A simple mention of 'ANOVA' and 'LSD post-hoc' does not say much about the analysis applied. Also reporting just p values is not informative.

We thank the reviewer for this remark. We now state in the manuscript: "Changes in body weight in the irradiated group given vehicle solution (irradiated control group) were compared to changes in the irradiated groups treated with G. kola extract or vitamin C, and to changes in the non-irradiated control group in each week post-treatment, using one-way ANOVA followed by Fisher's least significant difference (LSD) post hoc test. Differences in EPM performances at the end of the treatment were also compared between G. kola- and vitamin C-treated irradiated groups, the non-irradiated control group, and the irradiated control group using one-way ANOVA followed by LSD test."

5. Given that the results have been plotted as mean with bars indicating SEM (as stated in methods), some p values are clearly confusing. For example in Figure 2B the comparison between control and dH_2O group has statistical significance ($P=0.002$) when the error bars from control group clearly reach the mean value of dH_2O group. This is impossible.

Authors thank the Reviewer for pointing out this error. The EPM data as mean ± standard deviation while changes in body weight were plotted as mean ± SEM. All data are now presented as mean ± standard deviation.

Exacerbated claims

6. Data presented here suggest that the clinical image of the animals which receive irradiation is very poor. They barely move, or groom their fur, and they reduce the amount they eat. Especially the last two phenotypes are well known sign of distress and pain for laboratory animal (and more specifically rodents). Given this, I would urge caution when making any claims about cognitive effects of this irradiation procedure. Virtually every behavioural task used in rodents, and specifically EPM, relies on ambulatory activity or the subjects. Therefore, the most parsimonious explanation of behavioural phenotypes after radiation poisoning is distress/pain that leads to reduced movement.

Irradiated animals displayed an almost normal exploratory behavior and social interactions while in cage. As well documented in the literature, the radiation intensity to which they were exposed and the exposure time do not suffice to induce signs of acute poisoning. This is also strongly supported by the increase in body weight observed in the irradiated control group. We observe that if our animals were able to “barely move, or groom their fur, and they reduce the amount they eat”, they would not eat enough to grow as observed in the gain in body weight. The experiment was stopped 9 weeks after exposure, that is before severe signs of toxicity were observed. We reported in the manuscript that at this point animals had “decreased exploratory activity and reduced

social interactions" (thus were moving and had no sign of pain). Furthermore, these animals were handled every 3 days to change cage litter and to perform EPM and none expressed the common laboratory rodent marker of pain vocalization during the handling period. This precision has been included in the revised manuscript.

Reviewer: 2

Comments to the Author

1. The manuscript titled *Garcinia kola* improves cognitive and motor function of a rat model of acute radiation syndrome in the elevated plus maze is well written and conceived but I have some on the use of EPM for cognitive decline, although, EPM could be used to measure cognitive functions but the parameters observed for in this study are basically anxiety-like and motor functions [Time spent in central platform could not be use as yardstick for memory function but that of decision making].

Authors agree with the Reviewer that the time spent in central platform is indicative of the quality of the decision-making ability of the animal. There is no mention of memory function in the manuscript. Instead, it was stated at the end of the third paragraph of the Discussion that ". An increase in central platform time, which typically indicates an impairment in animal's ability to choose the arm to explore [14, 15], was observed in (irradiated) animals"; (and thus, altogether EPM) "findings suggest that *G. kola* treatment prevented or mitigated the development of cognitive and motor impairment in gamma-irradiated rats."

2. the figures are too tiny thus, increase their sizes.

The sizes of the figures have now been increased.

3. it is necessary for the authors to assay for biochemical markers to ascertain the level of neurodegeneration having exposed the whole body to gamma irradiation, any impact on feeding.

The purpose of the present study was to assess the effects of *Garcinia kola* extract on cognitive and motor functions of a rat model of acute radiation syndrome. However, the authors agree with the reviewer that in a future study it would interesting to “ascertain the level of neurodegeneration having exposed the whole body to gamma irradiation” considering the paucity of data on this topic.

4. it will be advisable to expose only the head to radiation while shielding the rest of the body

We appreciate the Reviewer’s comment. Shielding the rest of the body would reduce the contribution of immune-to-brain and organ-to-brain signaling on the neurodegeneration, whose pivotal role in inflammation-mediated neurodegeneration was established by a score of reports. These mechanisms, which were elegantly shown in experimental LPS-induced neuroinflammation, cannot be removed from acute radiation syndrome (which will happen if the body was shielded).

Editorial Office comments:

1. The graphical abstract supplied has a lot of graphs and text that may not render well in our table of contents, could you please provide a simpler graphical abstract? A graphical abstract is a visual representation of the central finding or methodology of the paper. It will be displayed in the article and in the table of contents, and used to promote your article on Twitter. Authors should strive to make the graphical abstract informative, interesting, visually appealing, and straightforward (no caption needed, no abbreviations except gene/protein names).

The graphical abstract has been prepared and submitted as suggested.

2. Please upload it as a separate high resolution image file (tiff preferred). The technical requirements for graphical abstracts are:
   - -size of the submitted image: 1200 pixels square at 300dpi
   - -font of the text: Arial 12-16 points (smaller fonts will not be legible online)

The graphical abstract has prepared as suggested.

3. -Please include the page numbers corresponding to the items listed in the checklist.

Revisions have been made and highlighted in red on pages 5, 6, 8, 9, 17 and 18.

4. -Please include a paragraph about data availability in the 'Materials and Methods' section. We strongly encourage data sharing either as supplementary information or uploads to repositories which you can link in the paper.

Data will be added as supplementary information.
