# Supplementary material for: Garcinia kola improves cognitive and motor function of a rat model of acute radiation syndrome in the elevated plus maze
Source: Brain Commun. 2021 Jul 28;3(3):fcab170. doi: 10.1093/braincomms/fcab170 (PMC8361417; doi:10.1093/braincomms/fcab170)
Supplement: fcab170_Supplementary_Data [file Revised Manuscript Decision Letter.pdf › Revised Manuscript Decision Letter.pdf]

Dear Alfred,

It is a pleasure to accept your manuscript entitled "Garcinia kola improves cognitive and motor function of a rat model of acute radiation syndrome in the elevated plus maze" for publication in Brain Communications. You will see that the reviewer suggests the figures are too small, but they seem fine to me. We will see if the rest of the team agree and let you know if any further changes are needed at First look.

The next step is for your paper to go through "First Look". We will contact you again shortly if any final files or formatting changes are required for publication or if we have any queries.

If no changes are needed, your submission will be transferred to our Production Department and you will hear from the Production Editor in due course.

Video abstracts - Brain Communications encourages authors to provide a video abstract to accompany their article. These are hosted on the Brain/Brain Communications YouTube channel. If you are interested in producing a 5-min video abstract to promote your article, then please contact the Editorial Office (brcoms.editorialoffice@oup.com) for further details and guidelines.

Thank you very much for your contribution. On behalf of the Editors of Brain Communications, we look forward to your future contributions to the Journal.

Best wishes

Professor Tara Spires-Jones
Founding Editor, Brain Communications
Tara.Spires-Jones@ed.ac.uk

Reviewer(s)' Comments to Author:

Reviewer: 2

Comments to the Author
recommended for acceptance but the figures are too small
